# Lithological Inheritance Governs Spontaneous Vegetation Succession on Contaminated Soils and Indirectly Regulates Soil–Plant Uranium Transfer in High-Altitude Mine Wastelands, Southwest China

**DOI:** 10.3390/plants15060854

**Published:** 2026-03-10

**Authors:** Zhijun Wei, Yinquan Zhao, Linjun He, Guoyan Wang, Xinyu Hong, Kezhemo Ashuo, Sijian Zhou, Maoyuan Li

**Affiliations:** 1 College of Geography and Planning, Chengdu University of Technology, Chengdu 610059, China; 19138972344@163.com (Z.W.); wangguoyan@cdut.edu.cn (G.W.); 18111487181@163.com (X.H.); ashuokezhemo@163.com (K.A.); zhousijian@cdut.edu.cn (S.Z.); 15111852298@163.com (M.L.); 2 Institute of Ecological Resources and Landscape, Chengdu University of Technology, Chengdu 610059, China; hljddm@163.com; 3 State Key Laboratory of Geohazard Prevention and Geoenvironment Protection, Chengdu University of Technology, Chengdu 610059, China

**Keywords:** lithological inheritance, vegetation restoration, multi-element pollution, uranium sequestration, ANR

## Abstract

High-altitude mine wastelands in Southwest China present formidable challenges for ecological rehabilitation due to extreme climatic stressors and multi-element contamination. Ecological restoration is closely related to soil environment. However, the mechanism by which parent material-induced heterogeneity governs spontaneous vegetation succession is still poorly understood. We established 36 plots (216 quadrats) to investigate the soil physical and chemical properties and vegetation restoration of propylite, porphyry and siltstone in the Xifanping Copper Mine, Sichuan Province. Furthermore, fifteen metal/metalloid elements (Au, Ag, Mo, W, Cu, Pb, Zn, Hg, As, U, Se, Cr, Sn, Ti, Total Fe_2_O_3_), soil pollution and vegetation structure were evaluated. The study area exhibited severe composite pollution (mean Nemerow integrated pollution index = 8.09), primarily driven by Au, Ag, Mo, W, and Cu. Vegetation surveys identified 34 vascular plant species from 12 families. Propylite-derived substrates supported significantly higher species richness, Shannon–Wiener diversity, and soil organic matter than porphyry and siltstone. Redundancy analysis (RDA) identified soil organic matter (SOM) and bulk density (BD) as dominant environmental filters, with SOM explaining 14.03% of community variance (*p* < 0.01). Two native pioneers, *Potentilla supina* and *Cynoglossum wallichii*, were identified as specialized uranium (U) accumulators with bioconcentration factors of 13.39 and 4.49, respectively. Lithological inheritance dictates early successional trajectories by influencing edaphic structure and nutrient bioavailability. The identified U-accumulating species provide a valuable genetic resource for implementing Assisted Natural Regeneration (ANR) and developing sustainable phytoremediation strategies in contaminated alpine ecosystems.

## 1. Introduction

Mining activities are a critical driver of global industrial growth, yet they remain among the most ecologically disruptive human activities [1,2]. The extraction and processing of mineral resources frequently result in the loss of vegetation, removal of topsoil, and exposure of deep rock strata [3]. These processes disrupt pedogenesis and generate barren lands enriched in heavy metals and metalloids, often persisting as long-term ecological liabilities [1,4]. In China alone, mining has affected approximately 6.3 × 10^3^ km^2^ of land [4], creating a pressing need for effective rehabilitation and ecosystem reconstruction.

Vegetation restoration is an effective way to alleviate soil degradation and restore ecosystems in mining wastelands [5,6]. While artificial restoration can rapidly increase vegetation coverage in a short period of time [2,7], it also brings problems such as ecosystem instability [5,8]. Compared with artificial restoration, natural restoration of vegetation can form a more stable community structure [9,10,11] and integrate better into the local ecosystem [12]. This sustainable restoration technology [13] is listed as a priority development technology for forest restoration in China.

The natural restoration of vegetation in abandoned mine land is affected by many factors, such as climate, altitude, topography, slope, soil and so on [4,14]. Soil compaction, heavy metal toxicity and nutrient deficiency are the main abiotic factors restricting vegetation restoration [15,16]. For example, high soil bulk density and soil compaction lead to hardening and anaerobic conditions, which are not conducive to plant rooting [17], resulting in limited plant growth and development [12]. Soil organic matter, available phosphorus, available potassium and total nitrogen are positive factors for mine vegetation restoration and can promote vegetation restoration [18,19,20]. Different mine wastelands have different environmental conditions, and the key factors affecting the natural restoration of plants are not completely consistent. In alpine mining areas, low temperature and soil degradation are the main causes of limited plant growth and difficult ecological restoration [21,22,23]. In semi-arid areas, water is a key factor for mine vegetation restoration [24]. Soil phosphate, total nitrogen, and total organic carbon concentrations are major factors in the restoration of plant communities in the Jharkhand coal mining area, India [25]. In the coal gangue mining area of Shanxi, China, soil organic matter is the main reason affecting the natural restoration of vegetation [26]. Therefore, in order to accelerate the restoration of ecosystems, it is necessary to understand the abiotic limiting factors that affect the formation of plant communities under specific environmental conditions, as well as species composition and distribution [27], and develop more native plants to participate in degraded soil remediation [9,10,28].

In recent years, ecologists have increasingly recognized that the physical and mineralogical properties of parent materials—the so-called lithological inheritance—play a foundational role in shaping ecosystem development. This geological inheritance governs soil formation, influences nutrient dynamics, and ultimately drives vegetation community assembly. In high-altitude mining environments, where natural recovery is slow, lithology may act as the primary environmental template controlling successional direction and speed [29]. Despite this conceptual understanding, empirical evidence linking lithological inheritance to vegetation–soil feedbacks in contaminated high-altitude systems remains limited.

The Xifanping Copper Mine in Southwest China provides a unique natural laboratory for investigating lithology-driven ecological processes. The area features three distinct lithological substrates—propylite, porphyry, and siltstone—offering contrasting physical and chemical environments under otherwise similar climatic conditions [30]. This study aims to (1) evaluate the characteristics of heavy metal pollution in the soil of mining wasteland, (2) analyze how three specific parent rocks (propylite, porphyry, and siltstone) affect vegetation succession, and (3) explore which key soil factors will affect early natural vegetation restoration.

## 2. Results

### 2.1. Edaphic Heterogeneity and Pollution Patterns

The metal/metalloid mass fraction of the soil in the survey plot is shown in Table 1. The results showed that the contents of 15 target elements measured in the soil of the study area were higher than the background values of soil elements in Sichuan Province [31]. The single-factor pollution indices (S_i_) of Hg, Zn and U were 1.05, 1.60 and 1.69, respectively, indicating that these three elements in the study area were slightly polluted. As (2.91), Se (2.86), Total Fe_2_O_3_ (2.88), Ti (2.14), Cr (2.55) and Sn (2.29) indicated that these six elements belonged to the mild pollution level. Pb (4.54) was at a moderate pollution level; the single-factor pollution indexes of the other five metals—Au (7.10), Cu (5.51), W (7.25), Mo (8.11) and Ag (11.39)—were all greater than 5, indicating severe pollution. The Nemerow comprehensive pollution index (PI) in the soil of the study area reached 8.09, further indicating that the area was exposed to a severe pollution level.

**Figure 1:**
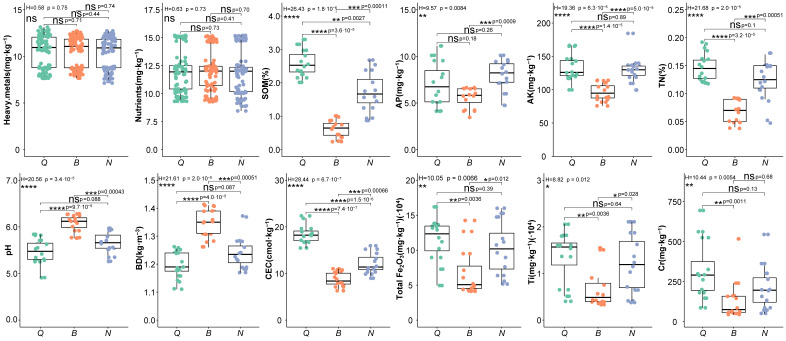
Difference analysis of physical and chemical indexes of three kinds of deep soil in the study area (‘Q’ represents the propylite sample plot; ‘B’ represents the porphyry plot; ‘N’ represents the siltstone sample plot; ‘Heavy.metals’ represents the content of heavy metal elements in soil (Au, As, Hg, Pb, U, W, Ag, Sn); ‘Nutrients’ represents the content of nutrient elements in soil (Se, Cu, Zn, Mo); * represents a significant level of *p* < 0.05, ** represents a significant level of *p* < 0.01, *** represents a significant level of *p* < 0.001, **** represents a significant level of *p* < 0.0001, ‘ns’ represents no significant correlation).

The soil developed by the three rocks is generally weakly acidic soil, and the pH value is between 5.47 and 6.07 (Figure 1). According to the classification standard of soil nutrient content in China’s second soil census, the SOM of the three soils was at a very low level (grade 6), and the AP content was medium (grade 4); the contents of TN and AK in propylitic and shaly sandstone soils were medium to high (grade 3), the TN content in porphyry soils was low (grade 5), and the AK content was medium (grade 4) (Chinese Soil Survey Office, 1998). Soil physicochemical properties varied significantly among lithologies. The pH value and BD of porphyry soil were significantly higher than those of the other two types of soil (*p* < 0.001), and the contents of TN and AK were significantly lower than those of the other two types of soil (*p* < 0.001). The content of AP followed the order porphyry soil < propylite soil < siltstone soil, and the content of AP in argillaceous sandstone soil was significantly higher than that of the other two types of soil (*p* < 0.001). The content of SOM and CEC followed the order porphyry soil < argillaceous sandstone soil < propylite soil, and the difference between the two was significant (*p* < 0.01). There was no significant difference in the contents of heavy metal elements such as Au, As, Hg, Pb, and U and nutrient elements such as Se, Cu, Zn, and Mo among the three soils. The content of Total Fe_2_O_3_ and Ti in porphyry soil was significantly lower than that in propylite and siltstone soils (*p* < 0.01), and the content of Cr was significantly lower than that in propylite soil (*p* < 0.05). Because 10 soil parameters were different in the three plots, these parameters were used in the correlation analysis between soil parameters and the species diversity index.

**Figure 2:**
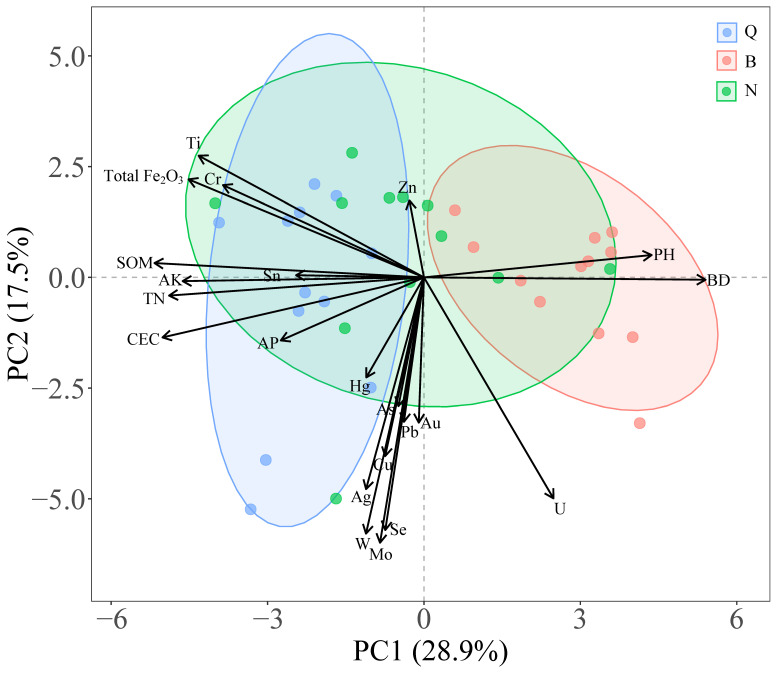
PCA analysis of soil physical and chemical properties of 36 plots in the Xifanping Copper Mine, Sichuan Province (‘Q’ represents the propylite sample plot; ‘B’ represents the porphyry plot; ‘N’ represents the siltstone sample plot).

Principal component analysis (PCA) was performed on soil physical and chemical properties across different plots (Figure 2). Among them, PC1 and PC2 explained 28.9% and 17.5% of the variation, respectively, and 46.4% of the total variation, indicating that the PCA can better reflect the overall differences in soil physical and chemical properties. On the PC1 axis, different types showed obvious separation characteristics. Porphyry plots were mainly distributed in the positive direction of PC1, which was significantly positively correlated with soil bulk density (BD) and pH. The propylite plot was mainly distributed in the negative direction of PC1, which was closely related to soil organic matter (SOM), total nitrogen (TN), available potassium (AK) and cation exchange capacity (CEC). On the PC2 axis, the differences among the sample plots were mainly affected by heavy metal elements. The siltstone sample had a strong correlation with heavy metal elements such as Zn, Hg, As, Pb, Au, Cu, Ag, Se, Mo and W, showing obvious metal enrichment characteristics. The propylite plot is relatively dispersed on the PC2 axis, and some of the samples are related to elements such as Ti, Cr and Total Fe_2_O_3_. In contrast, the distribution of porphyry plots on the PC2 axis is more concentrated, and the correlation with heavy metal indicators is weak.

### 2.2. Analysis of Plant Community Characteristics in the Study Area

#### 2.2.1. Community Composition and Stability

A total of 34 species of plants (Appendix A) were investigated in the study area, belonging to 12 families and 31 genera. Among them, Asteraceae (11 species, 32.35%), Lamiaceae (5 species, 14.71%), Polygonaceae (4 species, 11.76%), and Poaceae (3 species, 8.82%) accounted for 67.64% of the total species and were the dominant families in the study area. There were 30 plant species (88.24%) in propylitic soil, 25 plant species (73.53%) in siltstone soil and 22 plant species (64.71%) in porphyry soil. In total, 19 species of plants (55.88%) were distributed in all three plots, and these plants had strong adaptability. Seven species of plants (*Crotalaria yunnanensis*, *Clinopodium chinense*, *Isodon pleiophyllus*, *Ajania quercifolia*, *Geranium nepalense*, *Deyeuxia scabrescens*, *Bistorta vivipara*) were found only in propylitic soil. Two endemic plants (*Oxyria sinensis* and *Taraxacum mongolicum*) colonized only in siltstone soil, and two endemic plants (*Trifolium repens* and *Pedicularis gruina*) colonized only in porphyry soil.

**Figure 3:**
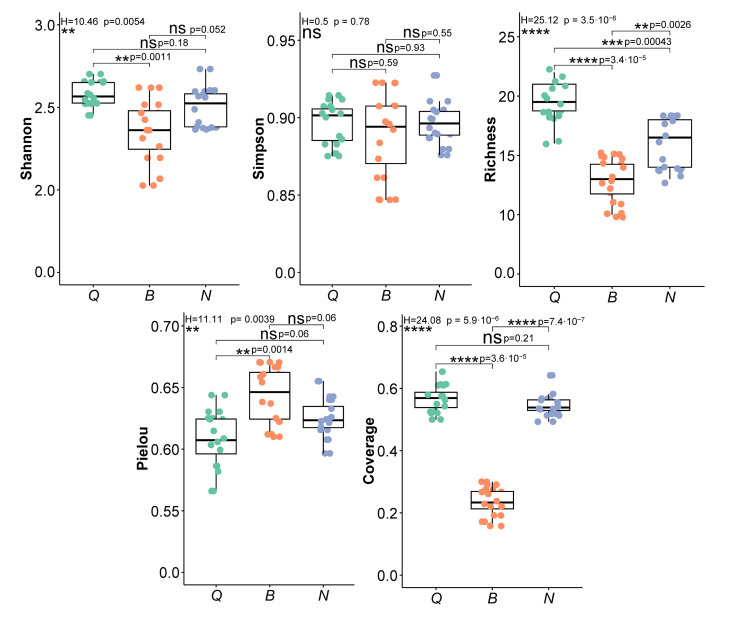
Comparative analysis of the plant diversity index of three parent rock plots in the study area (** represents significant level of *p* < 0.01, *** represents significant level of *p* < 0.001, **** represents significant level of *p* < 0.0001, and ‘ns’ represents no significant correlation).

Species diversity is an important indicator for measuring community structure and ecosystem stability [32]. After 5 years of natural restoration, the Patrick index, Shannon–Wiener index and Simpson index of plant community species in the three plots followed the order propylite > siltstone > porphyry (Figure 3). Among them, the Patrick index showed a significant difference (*p* < 0.001) and the Shannon–Wiener index also differed significantly between propylite and porphyry (*p* < 0.01). The Simpson index differed among the plots, but the difference was not significant. The Pielou index ranges from high to low for porphyry, siltstone and propylite, with a significant difference between porphyry and propylite (*p* < 0.01). The relative coverage of species was 0.567, 0.235 and 0.548, respectively. The relative coverage of species in the porphyry plot was significantly lower than that in propylite and siltstone (*p* < 0.001), and there was no significant difference between propylite and siltstone. There were significant differences in vegetation restoration among the three lithologic plots. Propylite and siltstone soils had relatively rich plant species and high coverage, while porphyry plots had fewer plant species and lower coverage.

After five years of natural restoration, the results of the community stability analysis of the three plots are shown in Table 2. The intersection points of the smooth curve simulation model equation and the linear equation of the three plots are 23.33/75.43, 31.82/68.7 and 29.42/69.64, respectively. The results show that the propylite plot is more stable than the other two types of plot communities (20/80 is the community stability point), suggesting stronger resilience and higher potential for successional stabilization.

#### 2.2.2. Analysis of Heavy Metal Absorption Capacity of Dominant Plants

**Figure 4:**
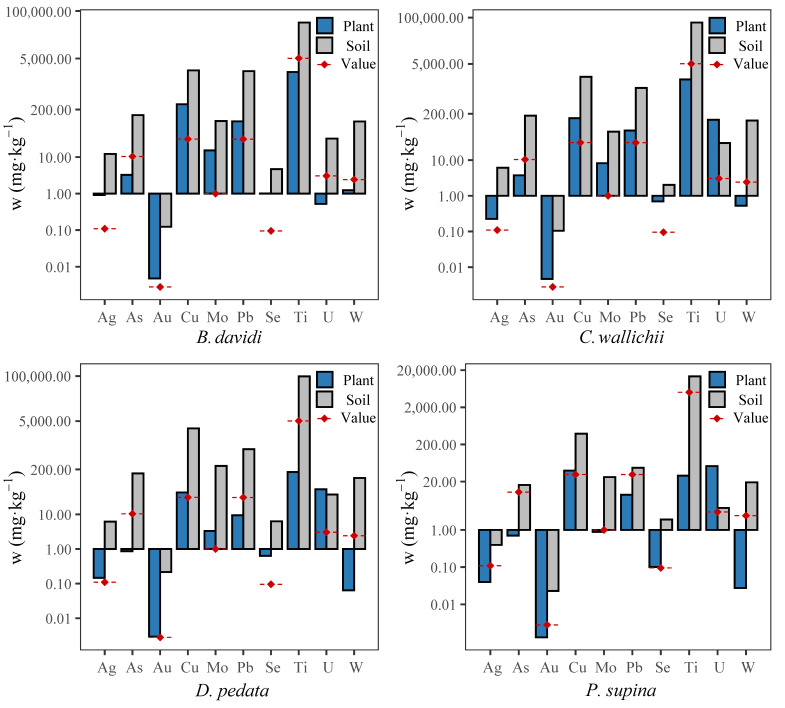
Statistical analysis of heavy metal mass fraction in dominant plants and rhizosphere soil in the study area (‘w’ represents the unit mass concentration of this element in plant, ‘Value’ represents the background value of soil elements in Sichuan; ‘*B. davidi*’ stands for *Buddleja davidi*, ‘*C. wallichii*’ stands for *Cynoglossum wallichii*, ‘*D. pedata*’ stands for *Dicranopteris pedata*, and ‘*P. supina*’ stands for *Potentilla supina*).

In this study, four dominant plants in three soil types were selected, including *Dicranopteris pedata*, *Cynoglossum wallichii*, *Buddleja davidi* and *Potentilla supina*. The contents of ten heavy metal elements in their tissues and their enrichment coefficients for different heavy metals were analyzed. The results of the element content analysis, shown in Figure 4, indicate that the contents of metal elements in soil are generally higher than those in plants, and are higher than the background values of soil elements in Sichuan Province. The contents of Cu, Pb, Ti and W in soil are relatively high, while the contents of Ag, Au and Se are generally low, with most being close to or lower than the background values.

**Figure 5:**
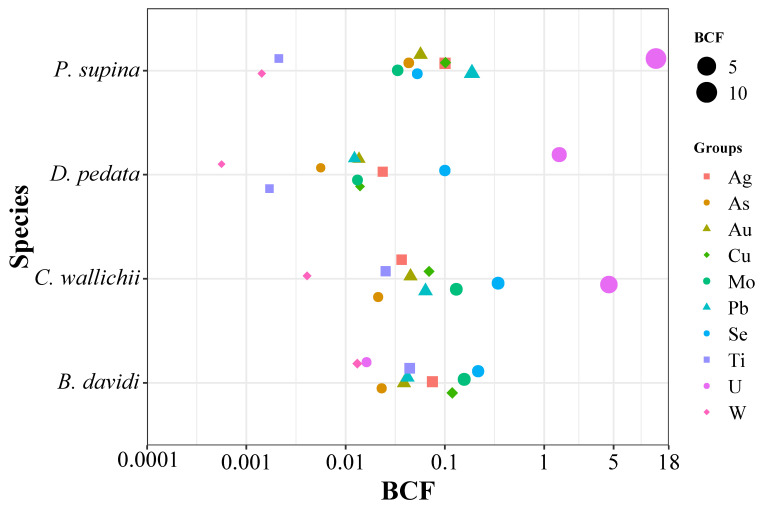
Enrichment factor (BCF) analysis of four dominant plant species in the study area (‘*B. davidi*’ stands for *Buddleja davidi*, ‘*C. wallichii*’ stands for *Cynoglossum wallichii*, ‘*D. pedata*’ stands for *Dicranopteris pedata*, and ‘*P. supina*’ stands for *Potentilla supina*).

The enrichment coefficients of the four dominant plants for different heavy metal elements are shown in Figure 5. The results show that the enrichment coefficients of heavy metals in *Buddleja davidi* are Se > Mo > Cu > Ag > Ti > Pb > Au > As > U > W, and the overall coefficients are less than 1. The trend of the enrichment coefficient of heavy metals in *Dicranopteris pedata*, *Cynoglossum wallichii* and *Potentilla supina* is roughly the same: U > Se > Ag > Cu > Au > Ti > W. The enrichment coefficients for the U element in the three plants are greater than 1: *Dicranopteris pedata* (1.42), *Cynoglossum wallichii* (4.49), and *Potentilla supina* (13.39), indicating that these three dominant plants have a strong absorption capacity for uranium (U).

### 2.3. The Relationship Between Soil Parameters and Species Diversity in the Study Area

**Figure 6:**
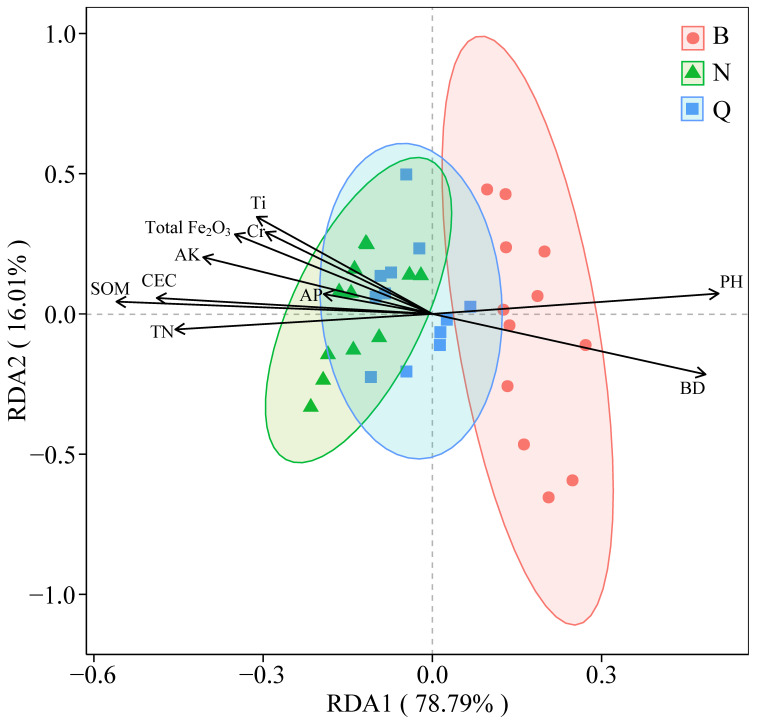
Redundancy analysis (RDA) of soil parameters and species diversity in the study area. (‘Q’ represents the propylite sample plot; ‘B’ represents the porphyry plot; ‘N’ represents the siltstone sample plot).

Decision Curve Analysis (DCA) was performed on the lithology-plant distribution-soil properties of the plot. The eigenvalues of the four single-peak ordination axes were less than 3 (0.10, 0.04, 0.05 and 0.04, respectively), and the RDA method was used for ordination. The results of the RDA analysis are shown in Figure 6. The first axis and the second axis explained 78.79% and 16.01% of the total variation in plant distribution in the study area, respectively. These two axes together explained 94.8% of the variation, indicating that environmental factors strongly influenced the differences among the plots. On the RDA1 axis, different lithologic plots showed obvious separation characteristics. Porphyry plots were mainly distributed in the positive direction of RDA1, which was significantly positively correlated with soil pH and bulk density (BD). The siltstone rock samples were mainly distributed in the negative direction of RDA1, which was closely related to soil organic matter (SOM), total nitrogen (TN), cation exchange capacity (CEC) and available potassium (AK). On the RDA2 axis, the degree of separation between the plots was relatively weak, but some samples showed a certain correlation with elements such as Ti, Cr and Total Fe_2_O_3_.

**Figure 7:**
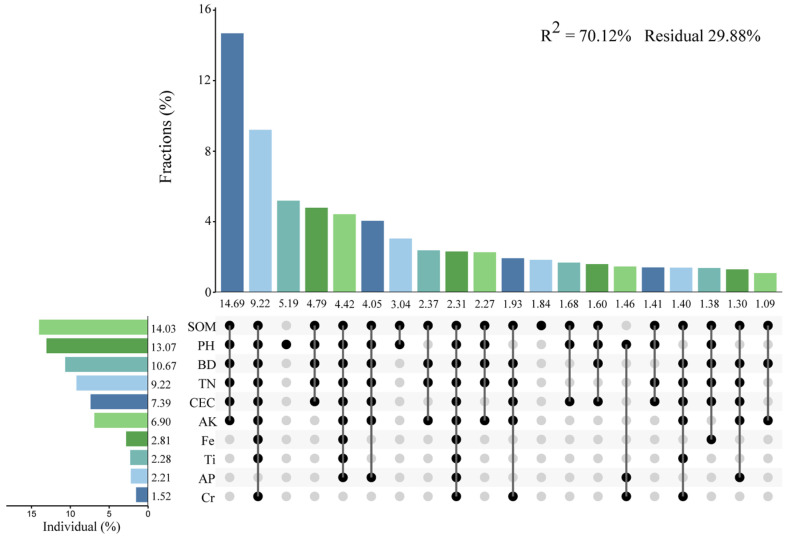
The contribution of different impact factors based on redundancy analysis to the overall contribution when they appear alone or in combination, The line represents the combined effect of multiple factors.

According to the above redundancy analysis (RDA) results, a hierarchical analysis was carried out (Figure 7). The results showed that the effects of different soil physical and chemical characteristics on community recovery were significantly different. SOM explained the most, at 14.03%, followed by soil pH (13.07%), soil BD (10.67%), TN (9.22%), CEC (7.39%), AK (6.90%), Total Fe_2_O_3_ (2.81%), Ti (2.28%), AP (2.21%), and Cr (1.52%). The interaction results of the different factors showed that SOM, soil pH, soil BD, TN, CEC and AK had the strongest interaction, accounting for 14.69%.

**Figure 8:**
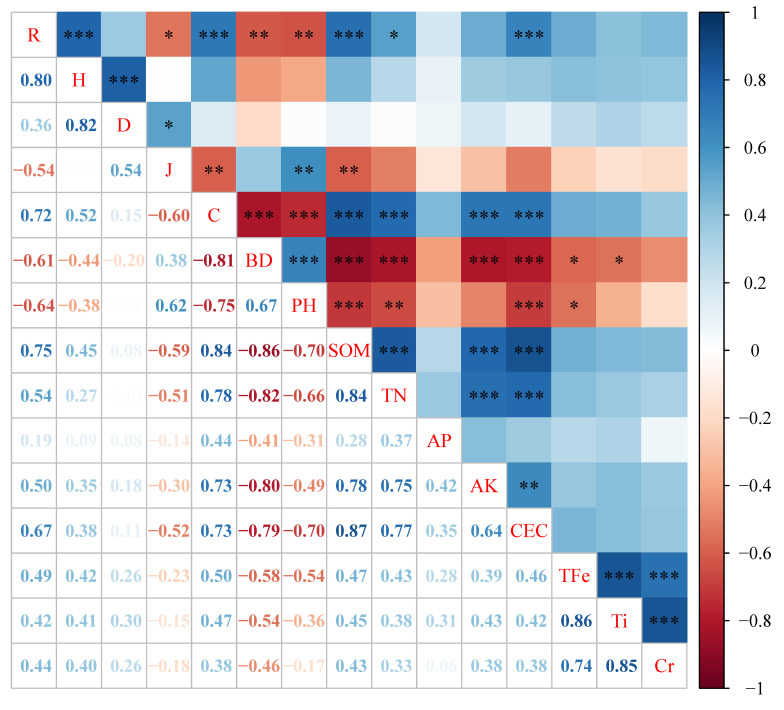
Correlation analysis between the species diversity index and soil factors in the Xifanping Copper Mine in Sichuan (* represents significant level *p* < 0.05, ** represents significant level *p* < 0.01, and *** represents significant level *p* < 0.001).

The correlation analysis between soil elements and species diversity (Figure 8) showed that the species richness index (R) was significantly negatively correlated with soil BD and pH (*p* < 0.01), and significantly positively correlated with SOM, TN and CEC (*p* < 0.05). The Pielou evenness index (J) was negatively correlated with SOM content (*p* < 0.01) and positively correlated with soil pH (*p* < 0.01). The relative coverage of species (C) was significantly negatively correlated with soil BD and pH (*p* < 0.001), and significantly positively correlated with SOM, TN, AK and CEC (*p* < 0.001).

## 3. Discussion

### 3.1. Effects of Soil Heterogeneity on Plant Communities

#### 3.1.1. Characteristics of Plant Communities in the Study Area

After five years of natural restoration, the study area has established 12 families, 31 genera and 34 species of plants. Among them, there are 11 species (32.35%) of Asteraceae, 5 species (14.71%) of Lamiaceae, 4 species (11.76%) of Polygonaceae, and 3 species (8.82%) of Poaceae, accounting for 67.64% of the total plant species. These families and genera are characterized by a large amount of seeds and easy dissemination, and have strong adaptability in the restoration of various mine wastelands such as coal mines and manganese mines [33].

The plant community in the study area is mainly composed of 1- and 2-year-old herbs and perennial herbs, with a ratio of about 1:2. Compared with the restoration of forest communities, the current community is still in the primary succession stage [34], and the life-form structure of plant communities is beginning to diversify. The analysis of community stability showed that the plant communities of the other two lithology and soil types were in an unstable state, except for propylite. Among them, the propylite soil plant community is stable and robust, which may be due to lower soil pH and BD values of the propylite soil and higher SOM, TN, CEC and AK than those of the other two soil types, making it more suitable for plant growth [35].

#### 3.1.2. Effects of Soil Heterogeneity on Plant Natural Restoration

Plant communities provide a large number of ecological services to the local area that help prevent soil erosion [36]. In China, natural restoration is a priority for forest ecosystem restoration, but the process of natural co-evolution of the soil–vegetation system takes a long time [3]. Through artificial active restoration measures, vegetation restoration in severely disturbed ecosystems can be accelerated [2].

This study shows that after 5 years of natural vegetation restoration in the abandoned land of a mineral exploration platform in the alpine region of Southwest China, the soil still shows obvious spatial heterogeneity and continuous high load characteristics of multi-element heavy metals. The general weak acidity (pH 5.47~6.07) is the common background of the soil in this area. The differences in pH, bulk density (BD), organic matter (SOM) and cation exchange capacity (CEC) caused by different lithologies may further affect the colonization and community construction of plants. It has been pointed out that soil pH is a key variable that controls the mobility, solubility and ecological risk of heavy metals. Especially in an acidification scenario, the mobility of metal ions is significantly enhanced, and plant colonization is more difficult [37]. This study further verifies the relationship between soil pH and heavy metal mobility. The results showed that higher soil pH reduced the bioavailability of metal ions, thereby reducing the absorption of these heavy metals by plants while also reducing their load on and toxicity to plants.

Porphyry soils in the study area showed higher pH and BD, while TN and AK were significantly lower, reflecting the limitations of soil compaction and barren soil, which hinder plant colonization and community stability. High BD usually means insufficient porosity and aeration, increased root penetration resistance and weakened water-nutrient transport efficiency, thereby inhibiting surface plant coverage and biomass accumulation [38]. In addition, low SOM and CEC reduce the adsorption and buffering capacity of metal ions and nutrients, and weaken the “chemical buffering—structural buffering” function of soil external disturbance, thus affecting the nutrient dynamics between plants and soil, and hindering the restoration of mining ecosystems. This combination of “high compaction—low SOM—low CEC” is widely regarded as a core limiting factor that hinders the establishment and subsequent succession of pioneer plants in mining wastelands [39].

The contents of various heavy metal elements detected in this study were significantly higher than the regional background values, and the single-factor pollution index and the comprehensive pollution index indicated a severe pollution level. Combined pollution usually acts through synergistic toxicity and nutritional imbalance. On one hand, Pb and As can interfere with plant photosynthetic and antioxidant systems; on the other hand, Cu and Zn can change from “essential trace elements” to “toxic stressors” at high concentrations and inhibit rhizosphere microorganisms, further weakening nutrient cycling and aggregate stability [29,40]. Recent reviews on heavy metal-contaminated soils also emphasize that long-term pollution affects the carbon and nitrogen cycles by changing microbial communities and key enzyme activities, thereby increasing the difficulty of vegetation restoration [29].

### 3.2. Effects of Soil Characteristics on the Absorption Capacity of Heavy Metals by Dominant Species

In this study, there were differences in the absorption capacity of four dominant species for different heavy metals. The average enrichment coefficient of *Buddleja davidi* was the lowest (0.074), and the average enrichment coefficient of *Potentilla supina* was the highest (1.397). This may be because different soil conditions have a direct impact on the accumulation and transport of metal elements in plants. The physical and chemical properties of soil affect the absorption, accumulation and enrichment ability of plants [41]. The discovery of *Potentilla supina* (BCF = 13.39) and *Cynoglossum wallichii* (BCF = 4.49) as high-capacity uranium accumulators provides new insights into the adaptive mechanisms enabling plant survival under multi-element stress. In acidic mine soils (pH 5.3–5.6), uranium primarily exists as uranyl ions (UO_2_^2+^), which are highly mobile and toxic to most plants. The strong accumulation of uranium in *P. supina* suggests an active uptake and detoxification mechanism rather than passive adsorption.

Soil pH is one of the key factors affecting the absorption of heavy metals by species. This study found that higher soil pH reduces the average enrichment coefficient of plants (Table 3), which may be because high pH leads to a decrease in the solubility of metal ions, thereby reducing the absorption by plants. For example, Kicińska et al. found that the solubility of metal ions in higher pH porphyry soils decreased, which inhibited the absorption of heavy metals by plant roots [37]. Changes in soil pH not only affect the mobility of heavy metals, but also may further change the tolerance of plants to heavy metals by affecting the structure and function of rhizosphere microbial communities [38]. Lower pH values usually increase the bioavailability of metals, thereby increasing the absorption capacity of plants; on the contrary, high pH reduces the availability of metals and the accumulation ability of plants [39]. Therefore, the regulation of soil pH is an important factor affecting vegetation restoration and heavy metal pollution management in mining areas. Adjusting the soil pH value can effectively improve the absorption capacity of plants and reduce the bioavailability of heavy metals. Properly adjusting the pH value and reducing the burden of heavy metals on plants may become an effective means to improve vegetation restoration in mining areas.

Moreover, the exceptional accumulation ability of *P. supina* and *C. wallichii* highlights their potential for assisted phytostabilization. The tolerance and bioaccumulation capacity of these species make them promising candidates for in situ remediation of uranium-contaminated soils while supporting natural vegetation recovery.

### 3.3. Relationship Between Soil Parameters and Species Diversity

The results showed that except for the J index, the plant diversity indexes H, D and R in propylite were higher than those in siltstone and porphyry soil (*p* < 0.05). Previous studies have shown that vegetation restoration in mining wastelands is closely related to soil physical and chemical parameters [11,20]. In this study, Pearson correlation analysis showed that plant diversity in the study area was highly correlated with soil physical and chemical parameters. However, soil AP was not correlated with J, R, H, D or other diversity indexes. RDA analysis showed that SOM accounted for the greatest proportion of vegetation restoration in the study area (14.03%). The correlation between SOM and multiple plant diversity indexes in the study area was between 0.59 and 0.86, with the highest correlation indicating that SOM played a key role in vegetation restoration in the study area. This study shows that there are significant differences in SOM among the three soil samples, which are mainly derived from soil parent materials [42], and the low pH environment of propylitic soil is also conducive to the accumulation of SOM [43].

As a reservoir of soil nutrients [44], SOM is the core index of soil quality. On the one hand, SOM can increase soil porosity and reduce BD [45], thereby improving soil water and fertilizer retention capacity; on the other hand, soil organic matter can regulate the dynamic balance of soil N, P, K and other elements, affecting species richness and diversity [34,46,47], which plays a key role in the ecological restoration of degraded soil. At the same time, SOM can also increase the available carbon source, promote the establishment of rhizosphere microorganisms and symbiotic bacteria, and indirectly enhance the nutrient acquisition ability of plants and their tolerance to metal stress. For example, the study of copper mine tailings by Gazitúa et al. shows that the local microbial community related to pioneer plants is a necessary condition for plant establishment, and the absence of microorganisms significantly reduces plant growth performance [48]. The study of tailings deposits by Gagnon et al. also showed that an increase in vegetation density would drive the active microbial community structure closer to that of natural soil [49]. These findings support the inference of this study that under the combined stress of heavy metals, “improving SOM and reconstructing microbial function” is an important pathway to promote the transition of the community from an early unstable state to a stable state.

In this study, pH was significantly negatively correlated with richness and coverage (*p* < 0.01). pH changes may change community structure in two ways: first, by changing the dissolution and adsorption equilibrium of potentially toxic elements, affecting bioavailability and plant toxicity exposure; second, by affecting nutrients (especially macro-elements such as P) and microbial activity, thereby reshaping competitive relationships [37]. In addition, BD was significantly negatively correlated with richness and coverage (*p* < 0.01), which is consistent with the general law of soil compaction limiting root growth. Relevant reviews have pointed out that compaction leads to a decrease in pores and an increase in penetration resistance, which significantly inhibits root elongation and nutrient absorption and reduces shoot growth [38]. Therefore, “SOM elevation—BD reduction—pH optimization—microbial recovery” should be regarded as different nodes of the same recovery path, rather than independent intervention targets [38,39]. In view of problems such as excessive heavy metals in the soil of the mining area, the combination of natural restoration and moderate artificial intervention can accelerate the vegetation reconstruction of a severely disturbed ecosystem [50], thereby better realizing the ecological restoration of secondary bare land. By optimizing soil physical and chemical properties, increasing SOM content and improving microbial community function, it can promote soil restoration in mining areas, improve vegetation restoration efficiency, and ultimately achieve stable and sustainable development of ecosystems.

### 3.4. Future Perspectives

Ecological succession in high-altitude mining ecosystems is inherently slow and strongly influenced by lithological and climatic constraints. While this study provides valuable insights into early successional stages, long-term monitoring is necessary to assess community dynamics and soil evolution over decadal timescales. Future research integrating metagenomic and transcriptomic approaches could reveal the microbial and genetic networks underlying metal sequestration and stress adaptation. Additionally, exploring soil amendments that mimic the beneficial properties of propylite substrates may offer practical strategies for accelerating ecological restoration in similar degraded environments.

## 4. Materials and Methods

### 4.1. Study Area and Quadrat Setting

**Figure 9:**
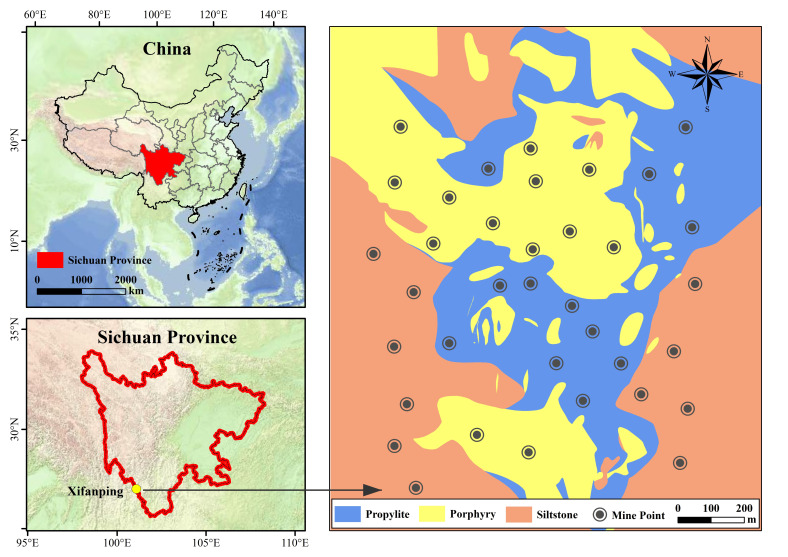
The schematic diagram of the Xifanping copper deposit in Sichuan Province (blue represents propylite, yellow represents propylite, orange represents siltstone, and black dot represents a mine point).

The study area is located in Yanyuan County, Sichuan Province, Southwest China, between 100°57′–101°00′ east longitude and 27°23′–27°27′ north latitude. The altitude of the study area is 2910~3150 m; the annual rainfall in the survey year (2024) was 2280.2 mm. The rainy season is from May to October, and the annual mean lower and higher temperature is −7.3 °C and 28.4 °C, respectively [51]. The study area belongs to the alpine forest landscape, and the forest vegetation is lush. There are evergreen coniferous and broad-leaved mixed secondary forests such as *Pinus yunnanensis*, *Quercus aquifolioides* and *Quercus glauca*. The bedrock types in the study area are mainly propylite, porphyry and siltstone. The excavation of the mineral exploration mountain leads to the destruction of the surface soil and vegetation, and the soil is completely exposed, forming a drilling platform. In 2019, the drilling platform was abandoned, and after 5 years of natural restoration of plants—in order to study the relationship between soil development and vegetation restoration in three parent rocks—12 platforms with an area of 90~130 m^2^ were selected as sample plots for each type of soil, resulting in a total of 36 sample plots (Figure 9). Because the altitude in the study area did not change much, the rain was sufficient during the growing season, and the site was a platform, the soil’s physical and chemical properties were used as explanatory variables in this study.

### 4.2. Vegetation Investigation

**Figure 10:**
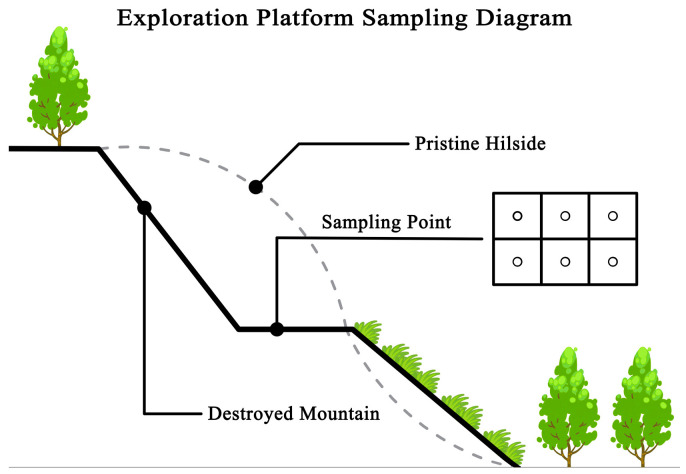
Sample plot of the exploration platform survey in the Xifanping Copper Mine in Sichuan.

In order to study the recovery status of the mining community, three profiles were set up in each plot along the direction from excavation to filling, and two plots of 1 × 1 m^2^ were set up in each profile. Each plot has 6 sampling points (Figure 10), resulting in a total of 36 plots and 216 sampling points. The plant species in each quadrat were investigated, and all species were identified at the species level. The species identification referred to the electronic version of «Flora of China» (http://www.iplant.cn/frps) (Editorial Board of Flora of China, Chinese Academy of Sciences, 2019). The crown width, height, coverage and total coverage of different species were measured, and the latitude, longitude and altitude of each plot were recorded by a hand-held GPS device.

### 4.3. Soil Sampling and Determination of Physical and Chemical Indexes

To collect undisturbed soil cores, the soil samples from 216 quadrats in 36 sample subplots were collected using the ring knife method, and the depth of collection was 0–20 cm. The surface soil (0~3 mm), plant roots, litter, etc., were removed. The soil samples from 6 sample points in the same plot were mixed evenly and placed in a bag at room temperature. Natural air drying, crushing, and screening with a 40-mesh sieve were conducted for soil physical and chemical analysis and testing. Referring to the method of Du et al. (2013), the soil pH was determined by the electrode potential method. Total nitrogen (TN) was determined by the semi-micro Kjeldahl method using a flow injection instrument. Available phosphorus (AP) was determined by 0.5 mol * L^−1^ NaHCO_3_ extraction followed by molybdenum antimony anti-color ultraviolet spectrophotometry. Available potassium (AK) was determined by NH_4_Ac extraction and atomic absorption spectrometry [46]. According to the method of Lu et al. (2000), the cation exchange capacity (CEC) was determined by the EDTA-ammonium salt rapid method, and the soil organic matter (SOM) was determined by the potassium dichromate bulk density method [52].

Soil As, Hg and Se were digested by aqua regia [53] and determined by atomic fluorescence spectrometry (AFS, AFS-3000, Hai Guang, Beijing, China). Other metals in the soil were digested by an HNO_3_-HF-HClO_4_ mixture [52], and the concentrations of Cr, Pb, Zn and Cu were determined by atomic absorption spectrometry (AAS, Solaar M6, Thermo Fisher Scientific, Waltham, MA, USA). The elements Ag, Au, U, Sn, Mo and W were determined by inductively coupled plasma mass spectrometry (ICP-MS, X Series II, Thermo Fisher Scientific, Waltham, MA, USA). Total Fe_2_O_3_ and Ti were determined by inductively coupled plasma atomic emission spectrometry (ICP-AES, iCAP6300, Thermo Fisher Scientific, Waltham, MA, USA). Soil reference material (GSS-5) and blank samples were used for quality control of each batch of soil samples, and the tests were repeated three times.

From July to August, when the plants grew vigorously, four dominant species—*Dicranopteris pedata*, *Buddleja davidi*, *Dicranopteris pedata* and *Potentilla supina*—were randomly selected from 36 plots. The whole plants were collected, and at least five plants of each species were collected and mixed into one group, resulting in a total of three groups. The plant samples were washed with ultrapure water, killed in an oven at 105 °C for 30 min, dried to a constant weight at 80 °C, and crushed through a 200-mesh sieve for use. The digestion and determination methods for the plant samples were the same as those for soil. Each batch of plant samples was subjected to quality control using a biological component analysis reference material (GBW10015) and a blank sample, repeated three times. The recovery of all standard samples was between 90.5% and 109.2%.

### 4.4. Data Statistical Analysis

#### 4.4.1. Soil Physical and Chemical Property Analysis

In order to explain the differences in physical and chemical indicators of the three distinct lithological substrates, the R (4.5.2) ggplot2 package was used to plot the soil physical and chemical indicators, the Wilcoxon rank sum test was used for pairwise comparison and the Kruskal–Wallis test was used for multiple comparisons. All soil data were normalized using log_10_(1 + X) for soil parameters.

#### 4.4.2. Species Diversity

Species diversity is an indicator of community structure and functional characteristics. In this study, the Partrick richness index (R), Shannon–Wiener diversity index (H), Simpson index (D) and Pielou evenness index (J) were used to represent the composition and spatial distribution pattern of species in the study area. Important values were used to describe the importance of species in different soil types. The calculation formula is as follows [44,54]:

Partrick richness index (1949):

(1) R = *S*

Shannon–Wiener index (H):

(2) $$H={-\sum_{i=1}^{s}P_{i}lnP}_{i}$$

Simpson index (D):

(3) $$D=1-\sum {(P}_{i}^{2})$$

Pielou evenness index (J):

(4) J= H/lns

Important value of species (IV) = (relative height + relative coverage + relative frequency)/3;

Relative coverage (%) = (the sum of the coverage of one species/the coverage of all species) × 100%;

Relative height (%) = (the height of a plant species/the sum of the individual heights of all species) × 100%;

Relative frequency (%) = (the sum of the frequency of a species/the frequency of all plants) × 100%;

‘*S*’ is the number of species observed in the plot, and ‘*P_i_*’ is the ratio of the i-th species to the total importance value of the community species.

#### 4.4.3. Community Stability Determination Method

The stability of plant communities in three types of plots was analyzed by the M. Godron method improved by Zheng Yuanrun [55]. The fuzzy scatter smoothing curve model

(5) y = a*x^2^* + b*x* + c

was established by taking the cumulative reciprocal percentage of species in the community as the abscissa (*x*) and the cumulative relative frequency of species as the ordinate (*y*). The straight line equation

(6) y = 100 − *x*

was substituted into (5) to obtain the intersection coordinate

(7) $$X=\frac{-(b+1)\pm \sqrt{{(b+1}^{)2}-4a(c-100)}}{2a}$$

In the formula, *a*, *b* and *c* are the parameters of the regression curve model. In the study, the solution of the equation 0–100 is taken (another solution is much larger than 100, which is an invalid value). The closer the *x*/*y* value is to 20/80 (the community stability point coordinates), the better the community stability is, and vice versa.

#### 4.4.4. Evaluation of Metals

In order to clarify the pollution status of single metal (class) elements in soil samples, the background value of soil elements in Sichuan Province was used as the evaluation standard. The single-factor pollution index method and the Nemero comprehensive pollution index method were selected for calculation and evaluation [56]. The calculation method and evaluation criteria are as follows:

(8) $$S_{i}=\frac{x_{i}}{B_{i}}$$

‘*S_i_*’ is the single pollution index of pollutants;

‘*X_i_*’ is the measured value of pollutants (mg·kg^−1^);

‘*B_i_*’ is the standard background value of pollutants. This paper uses the soil background value of Sichuan [31].

(9) $$PI=\sqrt{\frac{S_{j\cdot max}^{2}+S_{j\cdot av\mathrm{e}}^{2}}{2}}$$

‘*PI*’ is the Nemero comprehensive pollution index at the monitoring point;

‘*S_j.max_*’ is the maximum value of the single pollution index of all pollutants at the monitoring point;

‘*S_j.ave_*’ is the average value of the single pollution index of all pollutants at the monitoring point, and the evaluation classification standard and pollution degree are shown in Table 4 [57].

#### 4.4.5. Enrichment Coefficient of Heavy Metals in Plants

The accumulation ability of heavy metals in plants was evaluated by the bioconcentration factor (BCF) [58], which reflects the ability of plants to absorb heavy metals from the soil. The calculation formula is as follows:

(10) BCF = *W_p_*/*W_s_*

‘*W_p_*’ is the mass fraction of heavy metals in plants (mg·kg^−1^);

‘*W_s_*’ is the mass fraction of heavy metals in rhizosphere soil (mg·kg^−1^).

#### 4.4.6. Analysis of the Relationship Between Plant and Soil

Variables-PCA analysis was performed on the one-dimensional matrix constructed by plot-soil factors using the basic function prcomp () in the R (4.5.2) ggplot2 package to obtain the main factors affecting the differences in soil physical and chemical properties in the plot. In order to clarify the relationship between the distribution of species on different plots and soil factors, the decorana () function in the R (4.5.2) vegan package was used to perform the decision curve analysis (DCA) on the number of species in the study area. According to the DCA ranking results, canonical correspondence analysis (CCA) or redundancy analysis (RDA) was selected and the corresponding Monte Carlo test was performed. In order to understand the effects of different influencing factors and their interactions on community restoration, the R (4.5.2) psych and corrplot packages were used for correlation analysis, and the R (4.5.2) glmm.hp and upset.hp packages were used to intuitively understand the degree of interpretation of different influencing factors on community restoration.

## 5. Conclusions

In this study, we systematically analyzed the five-year natural restoration of vegetation in mountain mine wastelands in Southwest China and revealed the complex relationship between soil physical and chemical properties, heavy metal pollution characteristics and plant community diversity. The main conclusions are as follows:

The contents of various heavy metal elements (such as Pb, Cu, As, etc.) in the soil of the study area were significantly higher than the background values for Sichuan Province, and the soil was in a state of severe pollution. The combination of heavy metal pollution and low nutrient levels has become a major obstacle to vegetation restoration. There were significant differences in pH, bulk density, organic matter content and cation exchange capacity among the three lithological soils. Porphyry soil showed higher pH value and bulk density, with lower nutrient levels, limiting the recovery of vegetation. In contrast, the physical and chemical conditions of propylitic and siltstone soils are better, the vegetation coverage is higher, and the species diversity is richer.

The absorption capacity of *Buddleja davidi* is low, which is closely related to its high soil pH value. In this paper, it was found for the first time that the native plants *Potentilla supina* and *Cynoglossum wallichii* have a high enrichment effect on the U element, which provided a new choice for the remediation of soil U element pollution in mountainous mining areas.

The 34 species of plants are mainly annual and biennial herbs, and the community stability is low. Community diversity is closely related to soil organic matter, soil pH and bulk density. High organic matter content and suitable soil pH levels are conducive to vegetation restoration.

## Figures and Tables

**Table 1:** Statistical analysis of soil metal (class) element mass fraction and pollution index in the study area (‘S_i_’ is a single-factor pollution index; ‘PI’ is the Nemerow comprehensive pollution index; the ‘background value’ is the background value of soil elements in Sichuan Province that comes from ‘Background value of soil elements in China_1990’).

Index	Max/(mg·kg^−1^)	Min/(mg·kg^−1^)	Mean/(mg·kg^−1^)	CV(%)	Background Value	S_i_	PI
Au	0.11	0.001	0.02	14.49	0.0028	7.10	8.09
As	141.27	2.55	30.28	11.85	10.40	2.91	
Hg	0.17	0.02	0.07	3.30	0.065	1.05	
Se	0.59	0.03	0.27	9.51	0.095	2.86	
Cu	284.07	36.23	171.36	4.73	31.10	5.51	
Total Fe_2_O_3_	161,900.00	41,200.00	95,049.72	2.31	33,000.00	2.88	
Ti	21,093.00	3300.00	10,861.39	3.29	5072.00	2.14	
Cr	562.00	51.56	201.45	3.91	79.00	2.55	
W	49.80	2.49	17.54	7.05	2.42	7.25	
Zn	286.80	94.52	138.80	1.47	86.50	1.60	
Mo	35.57	1.10	8.11	7.34	1.00	8.11	
Pb	370.28	38.50	140.17	3.64	30.90	4.54	
U	8.83	3.14	5.16	1.64	3.05	1.69	
Ag	8.70	0.16	1.24	7.81	0.109	11.39	
Sn	7.86	2.34	5.26	2.25	2.30	2.29	

**Table 2:** The results of community stability analysis of three sample plots in the Xifanping Copper Mine in Sichuan (X and Y in the table represent the horizontal and vertical coordinates of the intersection of the smooth curve simulation model equation and the linear equation of the sample plot, respectively. ‘Relative stability’ indicates that the plot is in a relatively stable state, and ‘Instability’ indicates that the plot is in an unstable state).

Numbering	Plot Type	Curve Type	*R* ^2^	*p*	Intersection Point		Result
					X	Y	
Q	Propylite	y = 1.719X^2^ − 0.910X + 34.713	*R*^2^ = 0.931	<0.001	23.33	75.43	Relative stability
B	Porphyry	y = 1.874X^2^ − 0.018X + 16.889	*R*^2^ = 0.978	<0.001	31.82	68.76	Instability
N	Siltstone	y = 1.776X^2^ + 0.014X + 22.422	*R*^2^ = 0.956	<0.001	29.42	69.64	Instability

**Table 3:** Average soil pH and enrichment coefficient of dominant species in the study area.

Species	pH	BCF (Mean)
*Cynoglossum wallichii*	5.557	0.523
*Dicranopteris pedata*	5.593	0.160
*Buddleja davidi*	5.667	0.074
*Potentilla supina*	5.310	1.397

**Table 4:** Evaluation of the grading standards and pollution levels [57].

S_i_	Pollution Level	PI	Extent of Injury
S_i_ ≤ 1	Pollution-free	PI ≤ 0.7	Pollution free
1 < S_i_ ≤ 2	Slight pollution	0.7 < PI ≤ 1.0	Alert level
2 < S_i_ ≤ 3	Light pollution	1.0 < PI ≤ 2.0	Light pollution
3 < S_i_ ≤ 5	Moderate pollution	2.0 < PI ≤ 3.0	Moderate pollution
S_i_ > 5	Heavy pollution	PI > 3.0	Heavy pollution

## Data Availability

The original contributions presented in this study are included in the article. Further inquiries can be directed to the corresponding author.

## Appendix A

**Table A1:** Species composition table of the Xifanping Copper Mine in Sichuan.

Numb.	Scientific Name	Abb.	Lifestyle	Family	Propylite		Porphyry		Siltstone	
					Y/N	IV	Y/N	IV	Y/N	IV
1	*Erigeron annuus*	*E. annuus*	Annual herb	Asteraceae	1	0.98	1	0.81	1	0.47
2	*Galinsoga parviflora*	*G. parviflora*	Annual herb	Asteraceae	1	0.69	1	0.15	1	0.72
3	*Erigeron canadensis*	*E. canadensis*	Annual herb	Asteraceae	1	1.04	1	0.36	1	0.34
4	*Setaria viridis*	*S. viridis*	Annual herb	Poaceae	1	1.10	1	0.32	1	0.72
5	*Deyeuxia scabrescens*	*D. scabrescens*	Annual herb	Poaceae	1	0.36	0	—	0	—
6	*Polygonum dichotomum*	*P. dichotoma*	Annual herb	Polygonaceae	1	1.44	1	0.31	1	3.14
7	*Crotalaria yunnanensis*	*C. yunnanensis*	Annual herb	Fabaceae	1	0.4	0	—	0	—
8	*Cynoglossum wallichii*	*C. wallichii*	Biennial herb	Boraginaceae	1	5.68	1	1.94	1	4.11
9	*Artemisia scoparia*	*A. scoparia*	Biennial herb	Compositae	1	0.29	0	0	1	0.55
10	*Plantago asiatica*	*P. asiatica*	Biennial herb	Plantaginaceae	1	1.10	1	0.33	1	0.52
11	*Ajuga lobata*	*A. lobata*	Perennial herb	Lamiaceae	1	0.62	1	0.20	1	0.60
12	*Scutellaria forrestii*	*S. forrestii*	Perennial herb	Lamiaceae	1	0.03	0.20	0.20	0	—
13	*Elsholtzia densa* Benth.	*E. densa*	Perennial herb	Lamiaceae	1	0.30	0	—	1	0.25
14	*Dicranopteris dichotoma*	*D. pedata*	Perennial herb	Gleicheniaceae	1	2.35	1	0.50	1	1.15
15	*Taraxacum mongolicum*	*T. mongolicum*	Perennial herb	Compositae	0	—	0	—	1	0.31
16	*Kalimeris indica*	*A. indicus*	Perennial herb	Compositae	1	0.80	1	1.43	1	1.36
17	*Anaphalis nepalensis*	*A. nepalensis*	Perennial herb	Compositae	1	0.77	1	0.57	1	0.52
18	*Artemisia argyi*	*A. argyi*	Perennial herb	Compositae	1	0.83	1	1.19	1	0.86
19	*Rumex nepalensis*	*R. nepalensis*	Perennial herb	Polygonaceae	1	0.69	1	0.47	1	0.23
20	*Saussurea yunnanensis*	*S. yunnanensis*	Perennial herb	Compositae	1	0.28	0	—	1	0.45
21	*Pedicularis gruina*	*P. gruina*	Perennial herb	Scrophulariaceae	0	—	1	0.04	0	0
22	*Oxyria sinensis* Hemsl.	*O. sinensis*	Perennial herb	Polygonaceae	0	—	0	—	1	0.23
23	*Geranium nepalense*	*G. nepalense*	Perennial herb	Geraniaceae	1	0.04	0	—	0	—
24	*Trifolium repens*	*T. repens*	Perennial herb	Fabaceae	0	—	1	0.14	1	0.14
25	*Viola szetschwanensis*	*V. szetschwanensis*	Perennial herb	Violaceae	1	0.23	0	1	1	0.28
26	*Aster senecioides*	*A. senecioides*	Perennial herb	Compositae	1	0.33	1	0.26	1	0.87
27	*Clinopodium chinense*	*C. chinense*	Perennial herb	Labiatae	1	0.32	0	—	0	—
28	*Rabdosia pleiophylla*	*I. pleiophyllus*	Perennial herb	Labiatae	1	0.24	0	—	0	—
29	*Lolium perenne*	*L. perenne*	Perennial herb	Gramineae	1	0.16	1	0.31	1	0.38
30	*Fragaria moupinensis*	*F. moupinensis*	Perennial herb	Rosaceae	1	3.12	1	0.49	1	1.34
31	*Potentilla supina*	*P. supina*	Perennial herb	Rosaceae	1	2.28	1	1.63	1	2.57
32	*Polygonum viviparum*	*B. vivipara*	Perennial herb	Polygonaceae	1	0.06	0	—	0	—
33	*Buddleja davidi*	*B. davidi*	Subshrubs	Scrophulariaceae	1	2.44	1	0.93	1	2.11
34	*Ajania quercifolia*	*A. quercifolia*	Subshrubs	Compositae	1	0.28	0	—	0	—

Abbreviation = Abb.; Number = Numb.; Y/N indicates whether a species appears in a certain lithologic soil sample: Yes = 1, No = 0; Important value = IV; “—” Indicates that the species does not appear.
